# A polygenic resilience score moderates the genetic risk for schizophrenia

**DOI:** 10.1038/s41380-019-0463-8

**Published:** 2019-09-06

**Authors:** Jonathan L. Hess, Daniel S. Tylee, Manuel Mattheisen, the Schizophrenia Working Group of the Psychiatric Genomics Consortium, Rolf Adolfsson, Ingrid Agartz, Esben Agerbo, Margot Albus, Madeline Alexander, Farooq Amin, Ole A Andreassen, Maria J Arranz, Silviu A Bacanu, Steven Bakker, Gavin Band, Ines Barroso, Martin Begemann, Céline Bellenguez, Richard A Belliveau, Stephan Bender, Judit Bene, Sarah E Bergen, Elizabeth Bevilacqua, Tim B Bigdeli, Donald W Black, Hannah Blackburn, Jenefer M Blackwell, Douglas H R Blackwood, Elvira Bramon, Matthew A Brown, Richard Bruggeman, Nancy G Buccola, Randy L Buckner, Brendan Bulik-Sullivan, Suzannah J Bumpstead, Joseph D Buxbaum, William Byerley, Wiepke Cahn, Guiqing Cai, Dominique Campion, Rita M Cantor, Vaughan J Carr, Noa Carrera, Juan P Casas, Stanley V Catts, Kimberley D Chambert, Ronald Y. L. Chan, Raymond C. K. Chan, Eric Y. H. Chen, Wei Cheng, Eric F. C. Cheung, Siow Ann Chong, Sven Cichon, C. Robert Cloninger, David Cohen, Nadine Cohen, David A. Collier, Paul Cormican, Aiden Corvin, Nick Craddock, Benedicto Crespo-Facorro, James J. Crowley, Mark J. Daly, Ariel Darvasi, Michael Davidson, Kenneth L. Davis, Franziska Degenhardt, Jurgen Del Favero, Panos Deloukas, Ditte Demontis, Dimitris Dikeos, Timothy Dinan, Srdjan Djurovic, Enrico Domenici, Peter Donnelly, Gary Donohoe, Elodie Drapeau, Serge Dronov, Jubao Duan, Frank Dudbridge, Audrey Duncanson, Naser Durmishi, Sarah Edkins, Hannelore Ehrenreich, Peter Eichhammer, Johan Eriksson, Valentina Escott-Price, Tõnu Esko, Laurent Essioux, Ayman H. Fanous, Kai-How Farh, Martilias S. Farrell, Josef Frank, Lude Franke, Robert Freedman, Colin Freeman, Nelson B Freimer, Marion Friedl, Joseph I Friedman, Menachem Fromer, Pablo V. Gejman, Giulio Genovese, Lyudmila Georgieva, Eleni Giannoulatou, Ina Giegling, Michael Gill, Matthew Gillman, Paola Giusti-Rodríguez, Stephanie Godard, Jacqueline I. Goldstein, Vera Golimbet, Srihari Gopal, Jacob Gratten, Emma Gray, Hugh Gurling, Rhian Gwilliam, Lieuwe de Haan, Jeremy Hall, Christian Hammer, Naomi Hammond, Marian L Hamshere, Mark Hansen, Thomas Hansen, Vahram Haroutunian, Annette M. Hartmann, Garrett Hellenthal, Frans A. Henskens, Stefan Herms, Joel N. Hirschhorn, Per Hoffmann, Andrea Hofman, Mads V. Hollegaard, Hailiang Huang, Christina M. Hultman, Sarah E. Hunt, Masashi Ikeda, Nakao Iwata, Conrad Iyegbe, Assen V. Jablensky, Janusz Jankowski, Alagurevathi Jayakumar, Inge Joa, Erik G. Jönsson, Antonio Julià, Anna K. Kähler, René S. Kahn, Luba Kalaydjieva, Sena Karachanak-Yankova, Juha Karjalainen, David Kavanagh, Matthew C. Keller, Kenneth S. Kendler, James L. Kennedy, Andrey Khrunin, Yunjung Kim, George Kirov, Janis Klovins, Jo Knight, James A. Knowles, Bettina Konte, Vaidutis Kucinskas, Zita Ausrele Kucinskiene, Hana Kuzelova-Ptackova, Cordelia Langford, Claudine Laurent, Stephen Lawrie, S. Hong Lee, Phil Lee, Jimmy Lee, Sophie E. Legge, Todd Lencz, Bernard Lerer, Douglas F. Levinson, Cathryn M. Lewis, Tao Li, Qingqin S. Li, Miaoxin Li, Kung-Yee Liang, Jennifer Liddle, Jeffrey Lieberman, Svetlana Limborska, Kuang Lin, Don H. Linszen, Jianjun Liu, Jouko Lönnqvist, Carmel M. Loughland, Jan Lubinski, Milan Macek, Patrik K. E. Magnusson, Brion S. Maher, Wolfgang Maier, Anil K. Malhotra, Jacques Mallet, Hugh S. Markus, Sara Marsal, Ignacio Mata, Christopher G. Mathew, Morten Mattingsdal, Owen T. McCann, Robert W. McCarley, Steven A. McCarroll, Mark I McCarthy, Colm McDonald, Andrew M. McIntosh, Andrew McQuillin, Sandra Meier, Carin J. Meijer, Bela Melegh, Ingrid Melle, Raquelle I. Mesholam-Gately, Andres Metspalu, Patricia T. Michie, Lili Milani, Vihra Milanova, Younes Mokrab, Jennifer L. Moran, Derek W. Morris, Bryan J. Mowry, Bertram Müller-Myhsok, Kieran C. Murphy, Robin M. Murray, Inez Myin-Germeys, Benjamin M. Neale, Mari Nelis, Igor Nenadic, Deborah A. Nertney, Gerald Nestadt, Kristin K. Nicodemus, Liene Nikitina-Zake, Laura Nisenbaum, Annelie Nordin, Markus M. Nöthen, Eadbhard O’Callaghan, Michael C. O’Donovan, Colm O’Dushlaine, F. Anthony O’Neill, Sang-Yun Oh, Ann Olincy, Line Olsen, Roel A. Ophoff, Jim Van Os, Michael J. Owen, Colin N. A. Palmer, Aarno Palotie, Christos Pantelis, George N. Papadimitriou, Sergi Papiol, Elena Parkhomenko, Michele T. Pato, Carlos N. Pato, Tiina Paunio, Richard Pearson, Murray J Cairns, Lynn E DeLisi, Elliot S Gershon, Brian J Kelly, Max Lam, Nina Norgren, Sara A Paciga, Paul A Tooney, Jing Qin Wu, Lundbeck Foundation Initiative for Integrative Psychiatric Research (iPSYCH), Milica Pejovic-Milovancevic, Diana O. Perkins, Tune H. Pers, Tracey L. Petryshen, Olli Pietiläinen, Jonathan Pimm, Matti Pirinen, Robert Plomin, Andrew J. Pocklington, Danielle Posthuma, Simon C. Potter, John Powell, Alkes Price, Ann E. Pulver, Shaun M. Purcell, Digby Quested, Henrik B. Rasmussen, Anna Rautanen, Radhi Ravindrarajah, Abraham Reichenberg, Mark A. Reimers, Alexander L. Richards, Michelle Ricketts, Marcella Rietschel, Brien P. Riley, Stephan Ripke, Joshua L. Roffman, Panos Roussos, Douglas M. Ruderfer, Dan Rujescu, Veikko Salomaa, Alan R. Sanders, Stephen J. Sawcer, Ulrich Schall, Christian R. Schubert, Thomas G. Schulze, Sibylle G. Schwab, Edward M. Scolnick, Rodney J. Scott, Larry J. Seidman, Pak C. Sham, Jianxin Shi, Engilbert Sigurdsson, Teimuraz Silagadze, Jeremy M. Silverman, Kang Sim, Pamela Sklar, Petr Slominsky, Jordan W. Smoller, Hon-Cheong So, Erik Söderman, Chris C. A. Spencer, David St Clair, Eli A. Stahl, Elisabeth Stogmann, Amy Strange, Richard E. Straub, Eric Strengman, Jana Strohmaier, T. Scott Stroup, Zhan Su, Mythily Subramaniam, Patrick F. Sullivan, Jaana Suvisaari, Dragan M. Svrakic, Jin P. Szatkiewicz, Avazeh Tashakkori-Ghanbaria, Srinivas Thirumalai, Draga Toncheva, Sarah Tosato, Richard C. Trembath, Juha Veijola, Peter M. Visscher, Ananth C. Viswanathan, Damjan Vukcevic, John Waddington, Matthew Waller, Dermot Walsh, Muriel Walshe, James T. R. Walters, Qiang Wang, Dai Wang, Bradley T. Webb, Daniel R. Weinberger, Matthias Weisbrod, Mark Weiser, Jens R. Wendland, Paul Weston, Pamela Whittaker, Sara Widaa, Durk Wiersma, Dieter B. Wildenauer, Stephanie Williams, Nigel M. Williams, Stephanie H. Witt, Aaron R. Wolen, Emily H. M. Wong, Nicholas W. Wood, Brandon K. Wormley, Naomi R. Wray, Hualin Simon Xi, Clement C. Zai, Xuebin Zheng, Fritz Zimprich, Anders D. Børglum, Thomas D. Als, Jakob Grove, Thomas Werge, Preben Bo Mortensen, Ole Mors, Merete Nordentoft, David M. Hougaard, Jonas Byberg-Grauholm, Marie Bækvad-Hansen, Tiffany A. Greenwood, Ming T. Tsuang, David Curtis, Stacy Steinberg, Engilbert Sigurdsson, Hreinn Stefánsson, Kári Stefánsson, Howard J. Edenberg, Peter Holmans, Stephen V. Faraone, Stephen J. Glatt

**Affiliations:** 1 Psychiatric Genetic Epidemiology & Neurobiology Laboratory (PsychGENe Lab), Department of Psychiatry and Behavioral Sciences, SUNY Upstate Medical University, Syracuse, NY USA; 2 Department of Psychiatry, Yale New Haven Hospital, New Haven, CT USA; 3 iPSYCH, The Lundbeck Foundation Initiative for Integrative Psychiatric Research, Copenhagen, Denmark; 4 iSEQ, Center for Integrative Sequencing, Aarhus University, Aarhus, Denmark; 5 Department of Biomedicine - Human Genetics, Aarhus University, Aarhus, Denmark; 6 Bioinformatics Research Centre, Aarhus University, Aarhus, Denmark; 7 Institute of Biological Psychiatry, MHC Sct. Hans, Mental Health Services Copenhagen, Roskilde, Denmark; 8 Department of Clinical Medicine, University of Copenhagen, Copenhagen, Denmark; 9 National Centre for Register-Based Research, Aarhus University, Aarhus, Denmark; 10 Centre for Integrated Register-based Research, Aarhus University, Aarhus, Denmark; 11 Psychosis Research Unit, Aarhus University Hospital, Risskov, Denmark; 12 Mental Health Services in the Capital Region of Denmark, Mental Health Center Copenhagen, University of Copenhagen, Copenhagen, Denmark; 13 Center for Neonatal Screening, Department for Congenital Disorders, Statens Serum Institut, Copenhagen, Denmark; 14 Department of Psychiatry, University of California San Diego, La Jolla, CA USA; 15 University College London Genetics Institute, London, UK; 16 Centre for Psychiatry, Barts and the London School of Medicine and Dentistry, London, UK; 17 deCODE Genetics/Amgen, Reykjavik, Iceland; 18 Department of Psychiatry, National University Hospital, Reykjavik, Iceland; 19 Faculty of Medicine, University of Iceland, Reykjavik, Iceland; 20 Department of Biochemistry and Molecular Biology, Indiana University School of Medicine, Indianapolis, IN USA; 21 Medical Research Council Centre for Neuropsychiatric Genetics and Genomics, Department of Psychological Medicine and Neurology, School of Medicine, Cardiff University, Cardiff, UK; 22 Department of Neuroscience and Physiology, SUNY Upstate Medical University, Syracuse, NY USA; 23 Department of Public Health and Preventive Medicine, SUNY Upstate Medical University, Syracuse, NY USA

**Keywords:** Genetics, Schizophrenia

## Abstract

Based on the discovery by the Resilience Project (Chen R. et al. Nat Biotechnol 34:531–538, 2016) of rare variants that confer resistance to Mendelian disease, and protective alleles for some complex diseases, we posited the existence of genetic variants that promote resilience to highly heritable polygenic disorders1,0 such as schizophrenia. Resilience has been traditionally viewed as a psychological construct, although our use of the term resilience refers to a different construct that directly relates to the Resilience Project, namely: heritable variation that promotes resistance to disease by reducing the penetrance of risk loci, wherein resilience and risk loci operate orthogonal to one another. In this study, we established a procedure to identify unaffected individuals with relatively high polygenic risk for schizophrenia, and contrasted them with risk-matched schizophrenia cases to generate the first known “polygenic resilience score” that represents the additive contributions to SZ resistance by variants that are distinct from risk loci. The resilience score was derived from data compiled by the Psychiatric Genomics Consortium, and replicated in three independent samples. This work establishes a generalizable framework for finding resilience variants for any complex, heritable disorder.

## Introduction

Our progress in understanding the genetic basis for mental disorders has accelerated over the last decade due to improved methods and the increased sample sizes collated by the Psychiatric Genomics Consortium (PGC) [1]. Dissecting the genetic risk for these disorders is, in itself, extraordinarily valuable for guiding mechanistic studies, developing better diagnostics, and formulating therapeutics. But an understanding of risk states also has the benefit of allowing research on resilience. Knowing how some people avoid illness despite being at elevated risk should shed light on novel avenues for early intervention or treatment that could not be illuminated by studying affected individuals alone.

The psychological, sociological, and biological constructs of resilience, commonly defined as positive adaptation to extreme adversity [2], have been studied extensively. This paradigm of resilience focuses on “active coping” mechanisms; e.g., high emotionality, flexibility of thinking, having social support, and a sense of purpose, among others [3]. The way we are conceptualizing resilience differs from the traditional view in that we attribute resilience in part to heritable variation that increases resistance to disease, which closely relates to the paradigm that was invoked by the Resilience Project [4]. This new paradigm of genetic resilience focuses on the discovery of genetic variants that help unaffected individuals cope with a relatively large genetic burden of disease-associated variants. Our model has two postulates: (1) genetic resilience is in part mediated by common genetic variants that act by lowering the penetrance of risk variants, and (2) resilience variants are orthogonal to risk loci. The meaning of the second postulate is that resilience as defined here is not simply the inverse of risk. As opposed to “protective” variants, which are simply the alternate alleles at each risk-associated locus that have higher frequency in controls than cases, resilience alleles are hypothesized to ameliorate the effects of the risk loci and reduce the likelihood of the disorder. Research on the genetic basis of resilience (i.e., resistance to onset of illness) is contingent upon and necessarily lags behind the discovery of bona fide risk states. For schizophrenia (SZ), the Psychiatric Genomic Consortium (PGC) has identified a “polygenic risk score” that accounts for ~20% of the heritability on the observed scale (or ~6–7% on the liability scale) in risk through the additive effects of thousands of common variants [5, 6]. The reliability of this risk score continues to increase as additional samples contribute to its derivation; however, the genetic variance accounted for by additive effects of individual alleles appears to have recently reached an asymptote [7]. With the allelic-additive common-variant landscape of SZ coming into view, we have arrived at a point where a systematic and risk-informed study of the genetic basis for resilience to SZ is both possible and warranted, capitalizing on this estimate of polygenic risk.

The pursuit of genetic resilience factors for complex neuropsychiatric disorders is nascent, but not unprecedented. For example, the *APOE* ε2 allele is a well-known protective factor for Alzheimer’s disease that has been studied for its effects on biological and psychological features that may insulate carriers from the risk for the disorder. Results from prospective studies of psychiatric disorders related to trauma exposure, such as post-traumatic stress disorder (PTSD) in U.S. Marines who experienced extreme combat stress, suggest that genetic resilience factors may mitigate environmental vulnerability to mental illness [8]. Furthermore, studies of genetically modified mice shed light on promising candidate genes involved in glutamatergic synaptic transmission that increase resilience to phenotypes related to SZ [9]. The first bona fide study of genetic resilience was published by Chen et al. [4], which focused on rare diseases that manifest in childhood, and emerged from the Resilience Project undertaken by Mount Sinai and Sage Bionetworks [4]. Although our study is completely separate from the work by the Resilience Project, we consider both branches of work to parallel one another. In the Chen et al. [4], study, nearly 600,000 healthy adults were surveyed for highly penetrant mutations associated with Mendelian diseases that typically manifest during childhood (i.e., c.1558 G > T;p.V520F [cystic fibrosis], c.964-1 G > C [Smith-Lemil-Optiz syndrome], c.2204 + 6 T > T [Familial dysautonomia], etc.) [4]. The results from the Chen et al. [4] study suggest that a small number of individuals (~0.0022%) are genetically resilient to rare and devastating forms of childhood disease . Their study demonstrates that genetic resilience is an important avenue for understanding disease etiology. Investigating genetic and environmental factors that counteract inherited or exposed sources of adversity may help illuminate mechanisms that can be modulated to divert or reverse pathophysiological processes.

Here we present a general framework for identifying common variants that promote genetic resilience and for computing a “polygenic resilience score” that moderates the penetrance of known genetic risk factors. This is a proof-of-concept study that has the potential to increase understanding about the resilience to complex polygenic disorders. In essence, the strategy is to identify unaffected individuals at the highest levels of genetic risk, match them to affected individuals at equivalent levels of risk, and contrast these two subgroups to find residual variation associated with resilience. We present the results of applying this method in the largest available sample of SZ from the PGC and in three independent replication samples. We also describe general principles, specific parameters, limitations, and future applications of the approach, which may be useful for studying resilience to any heritable, complex, polygenic disorder.

## Methods

**Fig. 1:**
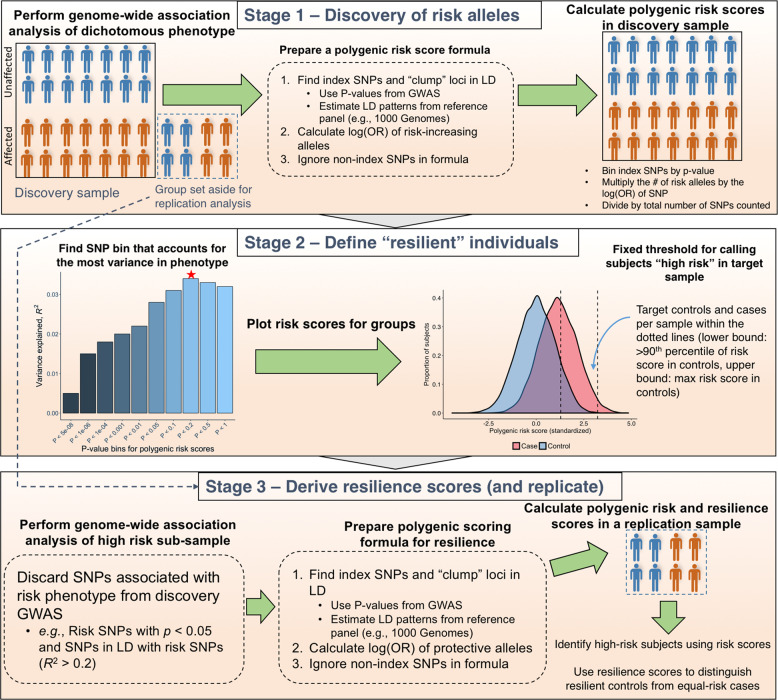
An Illustration of our method for deriving polygenic resilience scores for a complex disorder

Our approach to derive polygenic resilience scores for SZ is presented schematically in Fig. 1. Supplementary Table 1 outlines decision points that occurred in our analysis and parameters used at each point, including steps for truncating samples and variants for the GWAS and deriving an informative SNP set for resilience scoring. Wherever possible, we adhered to the precedent set by the PGC-SZ Working Group [5].

### Description of case–control GWAS samples

A description of sample ascertainment procedures used in each study is available in the supplementary text published by the PGC-SZ Working Group [5]. All subjects were confirmed to be independent based on relatedness tests using directly genotyped SNPs. Cases had a clinical diagnosis of SZ-spectrum disorders or SZ based on Diagnostic and Statistical Manual for Mental Disorder (DSM)-Version IV or International Classification of Diseases (ICD), 10th revision. Control ascertainment varied across studies. Our study inherited the same issues that the PGC-SZ faced in that some of the studies comprised control sets that were not screened for SZ, which to the best of our knowledge included the Gottingen Research Association for Schizophrenia (GRAS, controls *n* = 1232) and the Icelandic deCODE Genetics, Inc. sample which served as an out-of-sample replication set (controls *n* = 138,761). If there happen to be controls affected with SZ in our discovery sample (unlikely to be a large number as population prevalence of SZ is ~1%), then the loss of power potentially incurred in our analysis should be proportional to published GWAS by the PGC, which in that study was deemed reasonable [5].

### Meta-analysis of 51 GWASs in PGC2-SZ

We obtained GWAS summary statistics per study for the same 45 European-ancestry case–control studies, three East Asian-ancestry case–control studies, and three European trio-based studies assembled by the PGC Schizophrenia Working Group for their published wave 2 data set (PGC2-SZ) [5]. We then meta-analyzed single-variant association results genome-wide using inverse-variance fixed-effect summary statistics for these 51 studies using the inverse-variance fixed-effect method in the software *METAL* [10].

### LD-clumped SNPs and weights for risk scoring

We obtained from the PGC-SZ a list of 103,129 linkage-disequilibrium (LD)-independent SNPs and effect-size weights derived from the 51-study GWAS meta-analysis of SZ [5], along with imputed GWAS data for 45 of the 51 studies analyzed by Ripke et al. [5] (excluding six studies privately owned by pharmaceutical companies and unavailable to us for secondary analysis: four Johnson & Johnson and Roche samples, the Pfizer sample, and the Eli Lilly sample).

### Identifying subjects with high levels of polygenic risk

The variance in SZ explained by polygenic risk score (PRS) was maximized at a *p*-value bin of *p* < 0.05 [5], thus we used that threshold for selecting risk alleles for our PRS analysis. PRS were standardized using *z*-score scaling within each study. Subjects were then ranked by PRS and a percentile-based threshold was used to identify the highest-risk controls, i.e., “resilient” controls, along with cases with similarly high PRS. We set an arbitrary threshold at the 90th percentile of PRS in controls, and called controls above that threshold ‘resilient’ (different thresholds could be chosen). SZ cases whose PRS was between the 90th percentile for controls and the maximum PRS for controls were retained as the comparison group (Fig. 1). This method produced 3786 high-risk resilient controls (includes 83 pseudo-controls from trio studies) and 18,619 equal-risk cases (includes 494 cases from trio studies) for analysis. The total number of cases and controls retained per study after risk score matching is provided in Supplementary Table 2.

Ripke et al., reported that subjects in the highest PRS decile exhibit an increased risk for SZ (OR = 8–20) compared with the baseline rate of SZ in the lowest PRS decile [5], which is on-par with the estimated increase in risk for SZ among persons with affected first-degree relatives [11]. Building on these findings, Supplementary Table 3 shows that the population in the upper 10th percentile of PRS (who were included in our analysis) have an absolute risk of 40% and an increase in relative risk of 1.91 compared with all subjects above the lowest 10th percentile of PRS, which is similar to the relative increase in risk for those with an affected aunt or uncle [11].

### Derivation of polygenic resilience scores

Marginal SNP effects on resilience were computed using logistic regression models with *Plink* v1.9 [12] including adjustment for four principal components derived from autosome-wide SNP data that were significantly different between high-risk controls and equal-risk cases at a significance threshold of *p* < 0.1 to correct for population stratification (Supplementary Table 4). Any variant that showed an association with SZ risk from our meta-analysis of the 51 PGC2-SZ studies at a *p* < 0.05, or variants that were in LD with risk variants (at a *R*^2^ > 0.2 in a 1 megabase window), were discarded from this GWAS of resilience as a conservative measure, to avoid re-discovering risk variants and to ensure that any polygenic resilience score derived from our analysis was independent of the polygenic risk score used to stratify the samples. Our choice of threshold for pruning away SNPs in LD was used by the PGC and others [13–15]. We considered setting a stricter threshold of *R*^2^ ≤ 0.0, but this would have removed almost the entire genome and left only six variants for evaluation. Marginal SNP effects were obtained per study, and then pooled using inverse-variance fixed effect meta-analysis [10] to arrive at a final GWAS summary statistic per SNP.

Generating a polygenic score formula for resilience was done in a series of steps. Clumping the GWAS summary statistics output (*Plink* command: --clump-kb 500 --clump-r2 0.2 --clump-p1 1.0 --clump-p2 1.0) was performed using a reference panel that represents the predominant ancestry found in the GWAS sample (i.e., 1000 Genomes European phase 1 version 3, *n* = 379). We removed variants according to the following exclusion criteria referenced by the PGC-SZ working group in ref. [5] to retain an informative SNP set for polygenic resilience scoring: (i) variants in the MHC region (chr6:25 Mb-34 Mb), (ii) variants in the chromosome 8 inversion region (chr8:7 Mb–14 Mb), (iii) variants with an imputation quality score <0.9, and (iv) variants that are strand-ambiguous (AT/CG genotypes) or were small insertions/deletions. In addition, we removed variants with a minor allele frequency <0.05 and variants present in 10 or fewer studies. A total of 80,822 LD-independent SNPs associated with resilience to SZ were available for inclusion in calculating “resilience scores” in our training and test sets.

Resilience scores were determined by counting the number of protective alleles for sets of variants defined by *p*-value cutoffs (*p* < 0.0001, <0.001, <0.01, <0.05, <0.1, <0.2, <0.3, <0.5, <0.7, <1.0) and multiplying allele counts by the natural logarithm of the resilience odds ratio for each variant. We adopted a method used by the PGC [5] to estimate the amount of variance in resilience status that can be attributed to resilience scores. The approach is a two-model regression. In the first regression, resilience scores were specified as a predictor variable to estimate the odds of being a high-risk resilient control versus a matched-risk case (treated as the reference) per standard unit increase in resilience score. Four principal components derived from genome-wide SNP data that were significantly different between high-risk controls and high-risk cases were included in this regression as covariates to control for population stratification. A second logistic regression model was fit to estimate the amount of additive variance that the four principal component covariates that were specified in the first model contributed to resilience status. The variance in resilience status explained by each model was (based on Nagelkerke’s pseudo-*R*^2^) was calculated using the *R* package *fmsb* (version 0.6.1). We calculated the difference in *R*^2^ values between in the two models, yielding a value of *R*^2^ that was attribute to the amount of variance in resilience status explained by resilience scores.

### Correlation and interaction analyses of risk and resilience scores

We calculated Pearson’s correlation coefficients for risk and resilience scores in four separate groups: (1) SZ cases, (2) controls, (3) subjects with low-risk scores for SZ grouped by case–control status, and (4) and subjects with high-risk scores grouped by case–control status. Ultra-high-risk cases were excluded from the analysis (i.e., SZ cases with a risk score exceeding the maximum risk score of controls) as they lacked a matching set of controls. In addition, we performed a logistic regression analysis using all PGC samples (excluding ultra-high-risk cases) to test for a non-linear effect of risk and resilience scores on case–control status. Subjects were then split into deciles based on risk score. With logistic regressions, we computed the odds of a being a case between the bottom decile compared with each of the other deciles, as well as comparing the estimate the change in odds of being a control versus a case within each decile based on the unit increase in resilience score. The top 10 principal components for ancestry were included as covariates in the regression models.

### Replication

We had direct access to imputed GWAS data for two independent studies that were withheld entirely from all discovery analyses and used exclusively in our replication analysis (i.e., the Molecular Genetics of Schizophrenia (MGS), and the Danish iPSYCH study) along with the summary results from a third fully Icelandic sample from deCODE Genetics [16–18]. Subjects in the MGS (*n* controls = 2,482, *n* cases = 2,638) sample were ascertained from the US and Australia, which included cases with a DSM-IV diagnosis of SZ or schizoaffective disorder, and controls with no known history of mood, anxiety, substance use, psychotic, or bipolar disorders. Cases from the iPSYCH Consortium sample (*n* controls = 10,175, *n* cases = 3634) were ascertained from the Danish Civil Registration System and linked to the Danish Psychiatric Central Research Register to obtain diagnoses of SZ, whereas controls were ascertained by random sampling from the Danish Civil Registration System and removing individuals with a diagnosis of SZ or bipolar disorder. The third case–control replication set was the Icelandic population-based sample generated by deCODE Genetics, Inc [18]. comprised of 138,761 controls and 873 cases. Risk scores were calculated in the replication samples using SNP rsIDs and weights derived from the 51-study GWAS meta-analysis of risk. We applied our 90th percentile threshold method to identify high-risk controls and equal-risk cases. Resilience scores were calculated in the replication samples using the formulae derived in the discovery sample, and logistic regression models were used to estimate the effect of resilience scores on affection status and the proportion of variance in resilience explained by resilience scores after adjusting for select principal components to control for population stratification. For the MGS and iPSYCH samples, we selected principal components that significantly differed between high-risk controls and equal-risk cases at a significance threshold of *p* < 0.05 (three for MGS and two for iPSYCH). The top 10 principal components were used for the deCODE sample. We performed an inverse-variance fixed-effect meta-analysis with the *R* package *metafor* (version 2.0.0) using the natural logarithm of odds ratios and standard errors for resilience scores obtained from the MGS, Danish iPSYCH, and Icelandic deCODE samples, in order to assess the aggregate predictive capacity of resilience scores. Using code adapted from *Ricopili* (https://github.com/Nealelab/ricopili), the proportion of variance in resilience status explained by resilience scores was transformed to the liability scale assuming a population prevalence of 10% based on the 90% cutoff used to define resilience [19].

### Gene annotations

We downloaded a GTF file containing the positions of 57,820 protein- and non-coding genes, RNAs, and pseudogenes from the human reference genome version GRCh37.p13 from GENCODE [20]. The mapping of variants to genes was performed using the *R* package *GenomicRanges*. We retained annotations for protein-coding genes, non-coding genes (microRNAs, snoRNAs, snRNA, lincRNA), and antisense genes.

### Enrichment of resilience SNPs in risk genes

After annotating SNPs to genes, we computed association scores per gene for both risk and resilience to SZ by averaging the *z*-scores of intragenic SNPs within a given gene obtained from our meta-analyses. Gene scores were determined using 480,469 intragenic risk SNPs and 1,681,145 intragenic resilience SNPs. We did not extend the coordinates of genes beyond the intragenic region when mapping SNPs to genes. Only risk SNPs at a *p* < 0.05 significance level were included to ensure that risk SNPs were mostly LD-independent (*R*^2^ < 0.2) from resilience SNPs found within the same gene. A linear regression model was used to predict the per-gene risk association score using the per-gene resilience association score while simultaneously adjusting for gene length (in kilobases), the number of SNPs per kilobase of gene length, the average minor allele frequency of variants in each gene, and the chromosome the gene was located on. Cluster-robust standard errors were computed to correct for heteroscedasticity potentially caused by LD between genes.

## Results

### Polygenic resilience scores

**Fig. 2:**
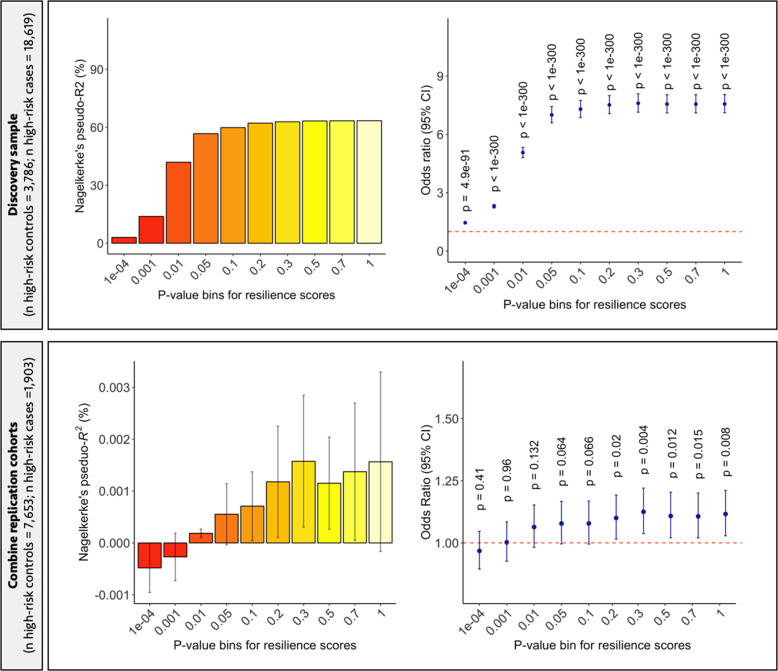
(Top panel) Polygenic resilience scores were computed in our discovery sample (high-risk controls = 3,786, high-risk cases = 18,619) based on results obtained from GWAS meta-analysis of resilience to SZ. The barplot shows the amount of variance in resilience explained by resilience scores (i.e., high-risk controls versus high-risk cases) explained by resilience scores across ten *p*-value bins. The dot plot shows corresponding Odds Ratios (OR) for resilience scores, wherein OR > 1.0 represents that high-risk controls have higher resilience scores compared with high-risk cases. (Bottom panel) The predictive performance of polygenic resilience scores is shown based on a meta-analysis of results obtained from three independent replication samples (Molecular Genetics of Schizophrenia, iPSYCH, and deCODE Genetics; high-risk controls = 7,653, high-risk cases = 1,903). Average Nagelkerke’s pseudo-*R*^2^ values calculated using arithmetic means and 95% confidence intervals are shown in the bottom left panel. Meta-analysis was used to pool natural log of OR and standard errors with an inverse-variance fixed effect model using the *R* package *metafor*

As expected, resilience scores were significantly associated with resilience status in our discovery set (top panel of Fig. 2) with a maximum of 46.9% of the variance in resilience explained by these scores (two-tailed *p* < 1.0 × 10^−300^). To replicate the association of resilience scores with resilience status, we performed a meta-analysis using results from three fully independent data sets: European ancestry subsample of the Molecular Genetics of Schizophrenia study (MGS; high-risk controls *n* = 244, high-risk cases *n* = 811), the Danish iPSYCH study (high-risk controls *n* = 931, high-risk cases *n* = 465), and Icelandic deCODE Genetics samples (high-risk controls *n* = 6944, high-risk cases *n* = 161). These samples yielded a total of 8,119 high-risk controls and 1,437 high-risk cases. Our meta-analysis replicated the significant association of resilience scores with resilience status across five *p*-value bins (*p* < 0.2, *p* < 0.3, *p* < 0.5, *p* < 0.7, and *p* < 1.0), with the most significant effect found at the *p* < 0.3 bin (OR = 1.12 per standardized unit increase in resilience score, SE = 0.041, two-tailed *p* = 0.0044; bottom panel of Fig. 2). The replicated effects of resilience scores survived multiple-testing correction for each *p*-value bin at the Benjamini-Hochberg FDR < 0.05 level. In the replication sets, resilience scores explained an average 0.042% (SD = 0.0001) of the variance in resilience status or 0.07% (SD = 0.0002) under the liability-threshold model (i.e., the SNP-heritability of resilience, or *h*^2^_*SNP*_).

Risk and resilience scores showed a small but significant positive correlation in the full discovery sample (Pearson’s *r* = 0.08, 95% CI = 0.068–0.083, degrees of freedom [*df*] = 66,615, two-tailed *p* < 8.2 × 10^−95^), but this differed by affection status. Risk and resilience scores were not significantly correlated among SZ cases (Pearson’s *r* = −0.003, 95% CI = −0.015–0.008, *df* = 28867, two-tailed *p* = 0.54), but were strongly and significantly positively correlated in controls (Pearson’s *r* = 0.47, 95% CI = 0.461–0.477, *df* = 37746, two-tailed *p* < 2.2 × 10^−16^), which validates the notion that, as risk score increases, so too must the resilience score in order for an at-risk individual to remain unaffected (Supplementary Fig. 3).

In the discovery sample, resilience scores were significantly higher in controls than cases (two-tailed *p* < 2.2 × 10^−308^), in high-risk controls than high-risk cases (two-tailed *p* < 2.2 × 10^−308^), and in high-risk controls than low-risk controls (two-tailed *p* < 2.2 × 10^−308^, Supplementary Table 5). Upon meta-analysis of the three replication samples, these difference in resilience scores replicated between controls and cases (two-tailed *p* = 0.013), high-risk controls and matched-risk cases (two-tailed *p* = 0.002), and high-risk controls and low-risk controls (two-tailed *p* = 8.3 × 10^−8^) (Supplementary Table 5). Resilience scores yielded significant associations only when higher *p*-value thresholds were used, thus we examined the level of LD between risk SNPs and resilience SNPs. We found a significant negative correlation between a SNP’s association with resilience and its level of LD with a risk SNP within 1 Mb (*r* = −0.032, *p* = 3.7 × 10^−17^), indicating that stronger resilience SNPs tend to have less LD with risk SNPs than weaker resilience SNPs. In addition, we found that the average LD between risk SNPs and resilience SNPs was low and relatively uniform across the ten *p*-value bins used to derive polygenic scores for resilience (range *R*^2^: 0.061–0.070). A significant negative interaction effect of risk and resilience scores on case–control status was found in the full PGC sample (*β* = −0.71, SE = 0.021, *p* = 1.6 × 10^−246^) in the presence of a significant main effect of risk score (*β* = 3.013, SE = 0.024, *p* < 1.0 × 10^−300^) and resilience score (*β* = −0.66, SE = 0.16, *p* < 1.0 × 10^−300^). As shown in Supplementary Table 6, an increased odds of being a case was found in higher deciles compared with the bottom decile of risk. Furthermore, controls showed significantly higher resilience scores compared with cases in the upper half of deciles (Supplementary Table 6).

### Top loci associated with resilience to SZ

**Fig. 3:**
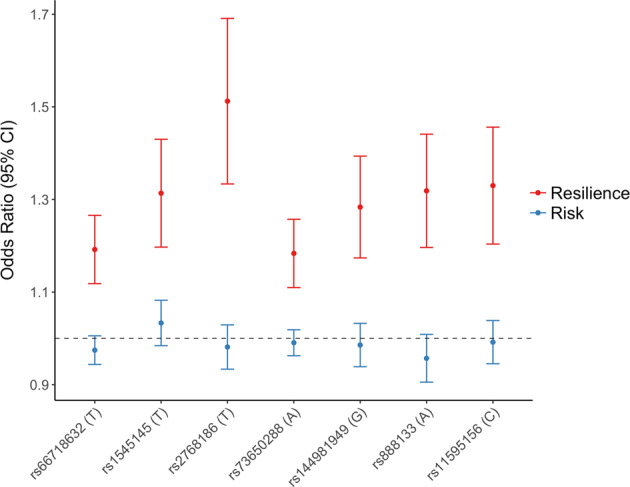
A pair of odds ratios and confidence intervals are plotted for the top seven common variants associated with resilience to SZ (*p* < 1× 10^−05^). This demonstrates that variants associated with resilience to SZ were not significantly associated with SZ risk (i.e., *p* > 0.05). Associations in red are for resilience obtained from the sample of individuals at the upper tail of the distribution of risk scores (i.e., high-risk controls, *n* = 3,775, and equal-risk cases, *n* = 18,581), whereas associations in blue were obtained from the full sample of SZ cases and controls in the published PGC-SZ2 data set (51 studies, *n* cases = 32,838, *n* controls = 44,357). A dotted line denoted no effect (i.e., OR = 1.0). An OR > 1.0 for red dots indicates that the allele was observed more frequently in high-risk controls than high-risk cases (i.e., increases resilience), whereas a OR > 1.0 for blue dots indicates that the allele was observed more frequently in cases than controls (i.e., increases risk)

Across the ~1.9 million variants (MAF ≥ 5%) examined in our meta-analysis, we compared the genome-wide resilience *p*-values with an expected (i.e., null) distribution of *p*-values, revealing that the observed values fit closely with expected values as shown in the quantile–quantile plot (Supplementary Fig. 1). The median ***χ***^2^ value from our GWAS deviated slightly from the expected *χ*^2^ value as given by the genomic inflation factor (i.e., *λ*_GC_ = 1.03), which was negligible compared with the level of inflation seen in the GWAS meta-analysis of SZ risk by (Ripke et al. [5]) (i.e., *λ*_GC_ = 1.468). None of the individual SNPs displayed a genome-wide significant association with resilience to SZ (Supplementary Fig. 2), which was not unexpected due to the small size of the subsamples of resilient controls and risk-matched cases relative to the full sample from which they were drawn. Results for the top seven resilience loci (*p* < 1.0 × 10^−5^) are provided in Supplementary Table 7. As shown in Supplementary Table 7, the top seven resilience SNPs exhibited low LD with risk SNPs (range *r*^2^ = 0.0063–0.089), confirming that top resilience loci are largely independent from risk loci. The magnitude of effect sizes for the top seven variants associated with resilience in the present analysis markedly exceeded effect sizes of those same variants on SZ risk in the full GWAS meta-analysis of SZ cases and controls (Fig. 3). One was nominally associated with risk in the full MGS replication sample (rs66718632, *p* = 0.035), and none in the other replication data sets.

We found qualitative evidence for this at the gene level as four of the top seven resilience SNPs and liberally defined risk SNPs (*p* < 0.05) map to the same genes but different LD blocks (Supplementary Table 8). A regression analysis of per-gene association scores for risk and resilience revealed a small but statistically significant positive linear relationship between a gene’s association with resilience and risk for SZ after adjustment for confounding variables described in the Methods section (regression *β* = 0.049, robust SE = 0.011, two-tailed *p* = 4.35 × 10^−6^).

## Discussion

Genetic analyses of resilience may help us understand factors that moderate the risk of mental illness among those at elevated risk. We present here a generalizable strategy to investigate genetic factors involved in resilience to complex polygenic disorders, as well as the first application of the method to demonstrate feasibility. SZ and several other psychiatric disorders have high heritability, and GWAS studies show that common variation is reliably associated with risk [1, 5, 21–27]. Our approach illuminates the role of common variation in buffering the penetrance of risk alleles associated with complex disease, and may aid in unraveling the sources of their etiologic heterogeneity. We assessed the validity of our method using the large publicly available genome-wide data assembled by the PGC for SZ and replicated both the direction of effect and the significance of a polygenic resilience score in three independent samples.

Our study produced the first estimate of the SNP-based heritability for resilience to SZ, which was relatively low (i.e., *h*^2^_*SNP*_ < 1%) compared with previous estimates of SNP-based heritability reported for SZ [5, 6]. Note that our approach did not look for resilience variants close to the risk variants, and so likely missed a portion. Deriving an estimate of the total heritability of resilience (i.e., *H*^2^) from family or twin-based studies will be important for interpretation of our SNP-heritability results, and for future studies that will explore the sharing of genetic predictors between resilience and other phenotypes. We showed that resilience scores produce conditionally independent effects on caseness relative to risk scores, confirming that our hypothesis that resilience alleles are not simply the inverse of the risk-associated alleles. Our resilience-scoring algorithm can be applied to GWAS data sets for other complex medical disorders, especially those for which polygenic risk scores have previously been derived in samples independent of those in which resilience is to be examined (such as our replication samples in the present study). These resilience scores can in turn be used to investigate heritable mediators of resilience, and potentially to identify factors that exert a risk-buffering effect across diagnostic boundaries. Cross-disorder meta-analyses of psychiatric GWAS data have already identified shared polygenic mediators of risk common to several disorders; this approach could be revisited in the context of resilience [22]. Another important question future studies may seek to address is: are there common genetic variants that influence resilience to SZ and also contribute to personality traits (i.e., extraversion, conscientiousness, openness, agreeableness) or constructs of cognitive well-being (i.e., educational attainment, genetic cognitive function) associated with the psychosocial construct of resilience? New methods could be devised to expand the capabilities of our approach or address shortcomings. For instance, because we could not derive a meaningful estimate of heritability from our results using LD score regression (LDSC) due to weak signals from regions of low LD, deriving new models for LDSC to allow for greater flexibility could be valuable. An alternative way to conceptualize resilience is as a form of gene–gene interaction wherein the penetrance of a risk variant can change based on the effect of a resilience variant. We modeled a relatively simple type of interaction by computing the multiplicative effect between risk and resilience score on case–control status and found that a significant interaction emerged between these scores (Supplementary Table 6). In future work, extensive simulation work could be performed to evaluate various types of interaction models to determine which contributes the strongest effects to resilience. Using knowledge about gene pathways and/or protein–protein interaction networks may help to discern epistatic effects between risk and resilience loci, and yield novel insight into the biology of disease. In short, there are multiple ways our method could be used and adapted to revisit previous single- and cross-disorder analyses to uncover genetic factors that mitigate vulnerability with shared or disorder-specific effects, and to account for the decreased penetrance and missing heritability in polygenic risk scores.

Because we focus on the tail of the risk score distribution, the sample size for our resilience analyses was an order of magnitude lower than the corresponding analyses of risk. Nevertheless, seven loci reached a ‘suggestive’ level of association (*p* < 1.0 × 10^−5^) with resilience to SZ. Assuming effect sizes hold in larger samples (despite the possibility of winner’s curse), adding about 2,030 high-risk controls to our analysis would be expected to yield sufficient power (>80%) to drive the lowest-ranking SNP in the 5.0 × 10^−8^ < *p* < 1.0 × 10^−5^ bin (e.g., rs11595156(T), OR = 1.32, allele frequency = 0.94) to genome-wide significance. However, it is important for independent replication in a much larger sample, which may be accomplished with the forthcoming phase-3 release of GWAS data from the PGC-SZ Working Group. Following replication, it will be appropriate to build on our genome-wide findings using bioinformatic tools, databases of molecular pathways, and functional annotations to identify and characterize the genetically driven biological pathways that mediate heritable effects on resilience.

To be conservative, we imposed some limitations on our approach, We used a conservative variant-filtering strategy to identify resilience-associated loci that were independent of risk variants. However, it is plausible biological that some resilience variants might be in the same regions or genes as risk variants. Risk-mediating common variants are more likely to occur in regions with broad LD [28], but the scope of the present study, after filtering out risk loci, restricted us to areas with relatively low LD. One caveat with our GWAS results was that resilience variants did not have strong LD support, because resilience variants were largely restricted to regions of low LD. Our approach was designed to identify resilience SNPs that are LD-independent of risk SNPs based on liberal definitions of risk (*p* < 0.05) and of LD (*R*^2^ < 0.2 with a risk-conferring variant) so that we avoided simply detecting additional risk SNPs; yet, biologically, it is expected that resilience SNPs can reduce the penetrance of nearby risk SNPs, even those within the same gene or LD block, such as by counteracting effects on gene expression levels (i.e., risk allele increases expression of gene while resilience allele decreases expression). Future work using Mendelian randomization or conditional association testing in much larger samples could be better suited to test the hypothesis that resilience signals are more likely to co-localize with loci and genes harboring risk variants for SZ.

There was a dramatic reduction in predictive performance of resilience scores when our model was applied to the much smaller, independent replication samples, as expected. Nevertheless, the resilience model was robust enough to yield significant prediction in the meta-analysis of replication samples. The sharp drop-off in variance explained indicates overfitting, which happens when training a model with an inadequate sample size or when too many noisy parameters are included in the model [29]. A challenge to our study design was statistical power, but we expect that the strength of our results in terms of variance explained and *p*-values of individuals SNPs will increase with the addition of more samples. The sample size for our study actually exceeded the discovery samples for recent PGC studies for anorexia nervosa [30], autism [21], and obsessive-compulsive disorder [31], as well the initial SZ study by the PGC in 2011 [32]. Looking back at the past decade of GWAS for SZ [5, 6, 26, 32, 33], our sample size is at the midpoint studies published between 2008 and 2009, and thus we might anticipate that, as in the study of SZ, the significance of individual resilience loci and their collective phenotypic variance explained will grow exponentially after the addition of more samples.

In conclusion, we have presented evidence for the validity of a method to identify individual loci and polygenic scores associated with resilience to a highly heritable complex disorder. This supports the idea that common variants that are not in LD with known SZ risk alleles exert a protective effect. Further replication will be key for validation of our method and findings. If our results are substantiated, for example in the forthcoming third wave of data from PGC-SZ, we anticipate a new wave of research on the genetics of resilience, the biology associated with resilience variants, and interventions that can foster resilience in at-risk populations.

## Supplementary information

Supplementary Materials

## Data Availability

Custom written *R* scripts used for statistical analyses can be provided upon request.

## Data availability

Genome-wide association summary statistics for all 1.9 million SNPs can be downloaded from: https://www.dropbox.com/sh/qhqbkcwvgzuwho3/AAClnqCvIdxIRsaklOLMGDLra?dl=0. The data used in this study were provided under restricted access by the Psychiatric Genomics Consortium (PGC), Lundbeck Foundation Initiative for Integrative Psychiatric Research (iPSYCH), and deCODE Genetics, Inc. Only summary statistics were made available to us from deCODE Genetics, Inc. We were granted access to imputed genome-wide SNP genotypes by PGC and iPSYCH. The data sets from PGC and iPSYCH remained on the Genetic Cluster Computer and GenomeDK cluster, respectively, where statistical analyses were performed.
