# Supplementary material for: A polygenic resilience score moderates the genetic risk for schizophrenia
Source: Mol Psychiatry. 2019 Sep 6;26(3):800–15. doi: 10.1038/s41380-019-0463-8 (PMC7058518; doi:10.1038/s41380-019-0463-8)
Supplement: Supplementary file 1 — Supplementary Materials [file 41380_2019_463_MOESM1_ESM.docx]

**Supplementary Materials**

**Title:** A Polygenic Resilience Score Moderates the Genetic Risk for Schizophrenia

**Authors**: Jonathan L. Hess^1^, Daniel S. Tylee^1^, Manuel Mattheisen^4-6^, the Schizophrenia Working Group of the Psychiatric Genomics Consortium^†^, Lundbeck Foundation Initiative for Integrative Psychiatric Research (*i*PSYCH), Anders Børglum^4-6^, Thomas D. Als^4-6^, Jakob Grove^4-7^, Thomas Werge^4,10,11^, Preben Bo Mortensen^4,5,12,13^, Ole Mors^4,9^, Merete Nordentoft^4,14^, David M. Hougaard^4,8^, Jonas Byberg-Grauholm^4,8^, Marie Bækvad-Hansen^4,8^, Tiffany A. Greenwood^15^, Ming T. Tsuang^15^, David Curtis^16,17^, Stacy Steinberg^18^, Engilbert Sigurdsson^19,20^, Hreinn Stefansson^18^, Kari Stefansson^18,20^, Howard J. Edenberg^21^, Peter Holmans^22^, Stephen V. Faraone^1,2^, and Stephen J. Glatt^1,2,3*^

**Affiliations:**

^1^ Psychiatric Genetic Epidemiology & Neurobiology Laboratory (PsychGENe Lab), Department of Psychiatry and Behavioral Sciences, SUNY Upstate Medical University; Syracuse, NY, USA

^2^ Department of Neuroscience and Physiology, SUNY Upstate Medical University; Syracuse, NY, USA

^3^ Department of Public Health and Preventive Medicine, SUNY Upstate Medical University, Syracuse, NY, USA

^4^ iPSYCH, The Lundbeck Foundation Initiative for Integrative Psychiatric Research, Denmark

^5^ iSEQ, Center for Integrative Sequencing, Aarhus University, Aarhus, Denmark

^6^ Department of Biomedicine - Human Genetics, Aarhus University, Aarhus, Denmark

^7^ Bioinformatics Research Centre, Aarhus University, Aarhus, Denmark

^8^ Center for Neonatal Screening, Department for Congenital Disorders, Statens Serum Institut, Copenhagen, Denmark

^9^ Psychosis Research Unit, Aarhus University Hospital, Risskov, Denmark

^10^ Institute of Biological Psychiatry, MHC Sct. Hans, Mental Health Services Copenhagen, Roskilde, Denmark

^11^ Department of Clinical Medicine, University of Copenhagen, Copenhagen, Denmark

^12^ National Centre for Register-Based Research, Aarhus University, Aarhus, Denmark

^13^ Centre for Integrated Register-based Research, Aarhus University, Aarhus, Denmark

^14^ Mental Health Services in the Capital Region of Denmark, Mental Health Center Copenhagen, University of Copenhagen, Copenhagen, Denmark

^15^ Department of Psychiatry, University of California San Diego, La Jolla, CA, USA.

^16^ University College London Genetics Institute, London, UK

^17^ Centre for Psychiatry, Barts and the London School of Medicine and Dentistry, London, UK

^18^ deCODE Genetics/Amgen, Reykjavik, Iceland

^19^ Department of Psychiatry, National University Hospital, Reykjavik, Iceland

^20^ Faculty of Medicine, University of Iceland, Reykjavik Iceland

^21^ Department of Biochemistry and Molecular Biology, Indiana University School of Medicine, Indianapolis, IN, USA.

^22^ Medical Research Council Centre for Neuropsychiatric Genetics and Genomics, Department of Psychological Medicine and Neurology, School of Medicine, Cardiff University, Cardiff, United Kingdom.

Table of Contents

[Supplementary Figure 1 3](#_Toc4761635)

[Supplementary Figure 2 4](#_Toc4761636)

[Supplementary Figure 3 5](#_Toc4761637)

[Supplementary Table 1 6](#_Toc4761638)

[Supplementary Table 2 7](#_Toc4761639)

[Supplementary Table 3 9](#_Toc4761640)

[Supplementary Table 4 10](#_Toc4761641)

[Supplementary Table 5 11](#_Toc4761642)

[Supplementary Table 6 12](#_Toc4761643)

[Supplementary Table 7 13](#_Toc4761644)

[Supplementary Table 8 14](#_Toc4761645)


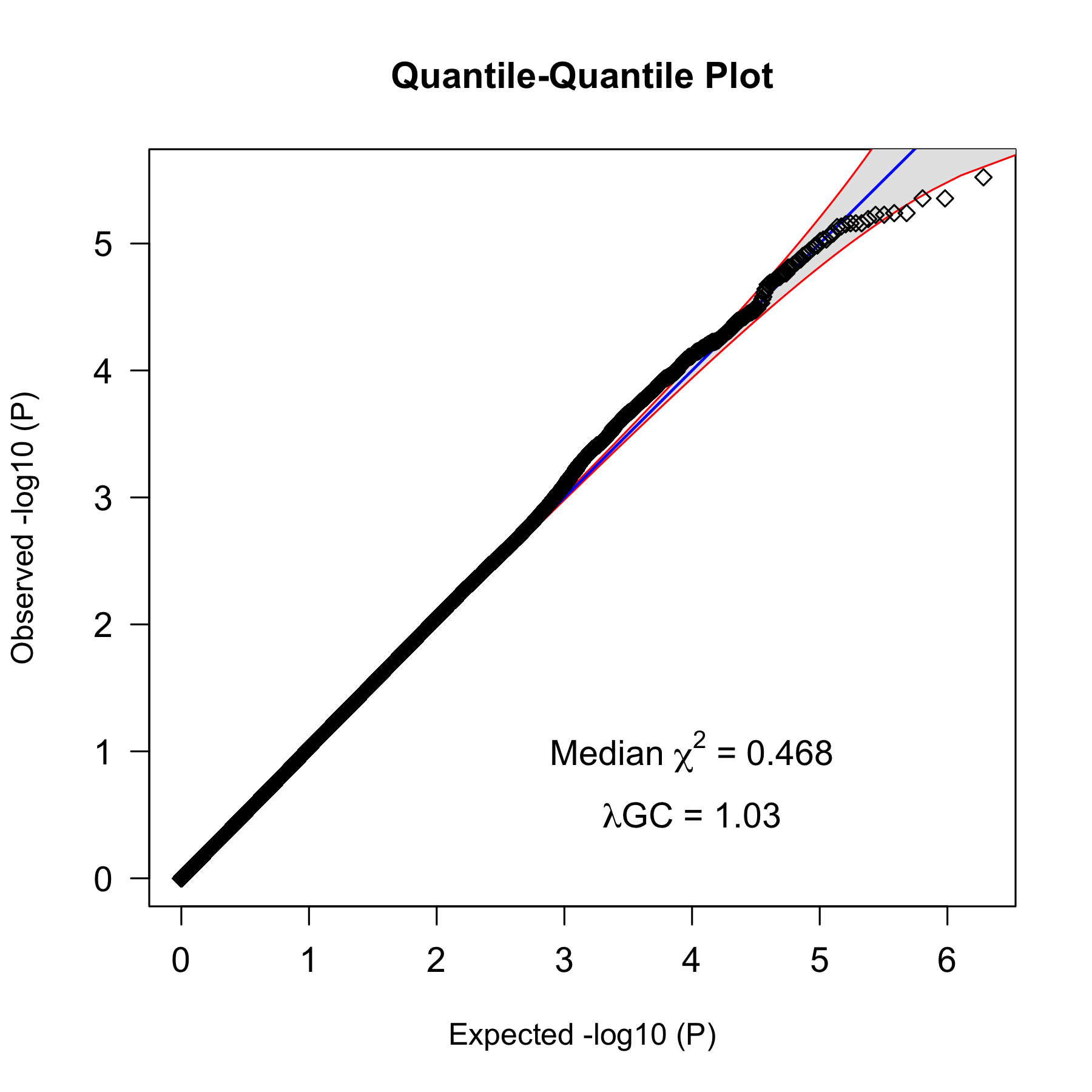


Supplementary Figure 1. A quantile-quantile (QQ) plot showing the distribution of p-values from our genome-wide association study meta-analysis of resilience to SZ (black diamonds) compared to a theoretical distribution of p-values for an equivalent number of variants (blue diagonal line).


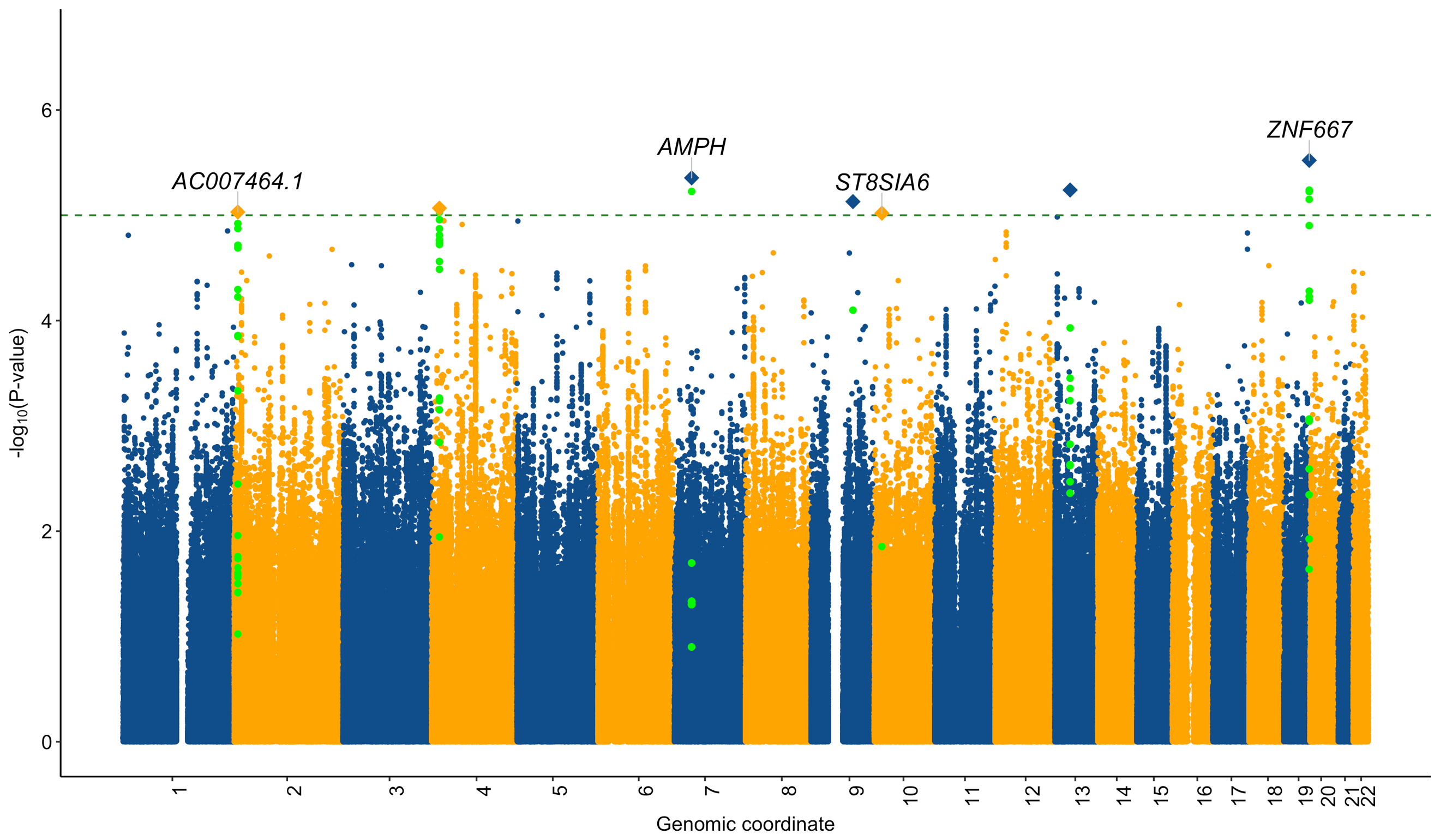


Supplementary Figure 2. A Manhattan plot showing genome-wide association results for our analysis of resilience to SZ for ~1.9 million common variants in autosomes (European minor allele frequency ≥ 5%). A horizontal dotted green line denotes a “suggestive” level of association for GWAS (*i.e.*, *p* ≤ 1×10^-5^). Lead index variants appear as diamonds (with *p* ≤ 1×10^-5^) and non-index variants are shown as dots. Non-index variants in linkage disequilibrium (*R*^2^ > 0.2) with index variants are shown in green. Index variants were labeled with HGNC gene symbols if the variant mapped in the transcribed region of a gene (*i.e.,* between the 5ʹ-untranslated region [UTR] and 3ʹ-UTR ends).


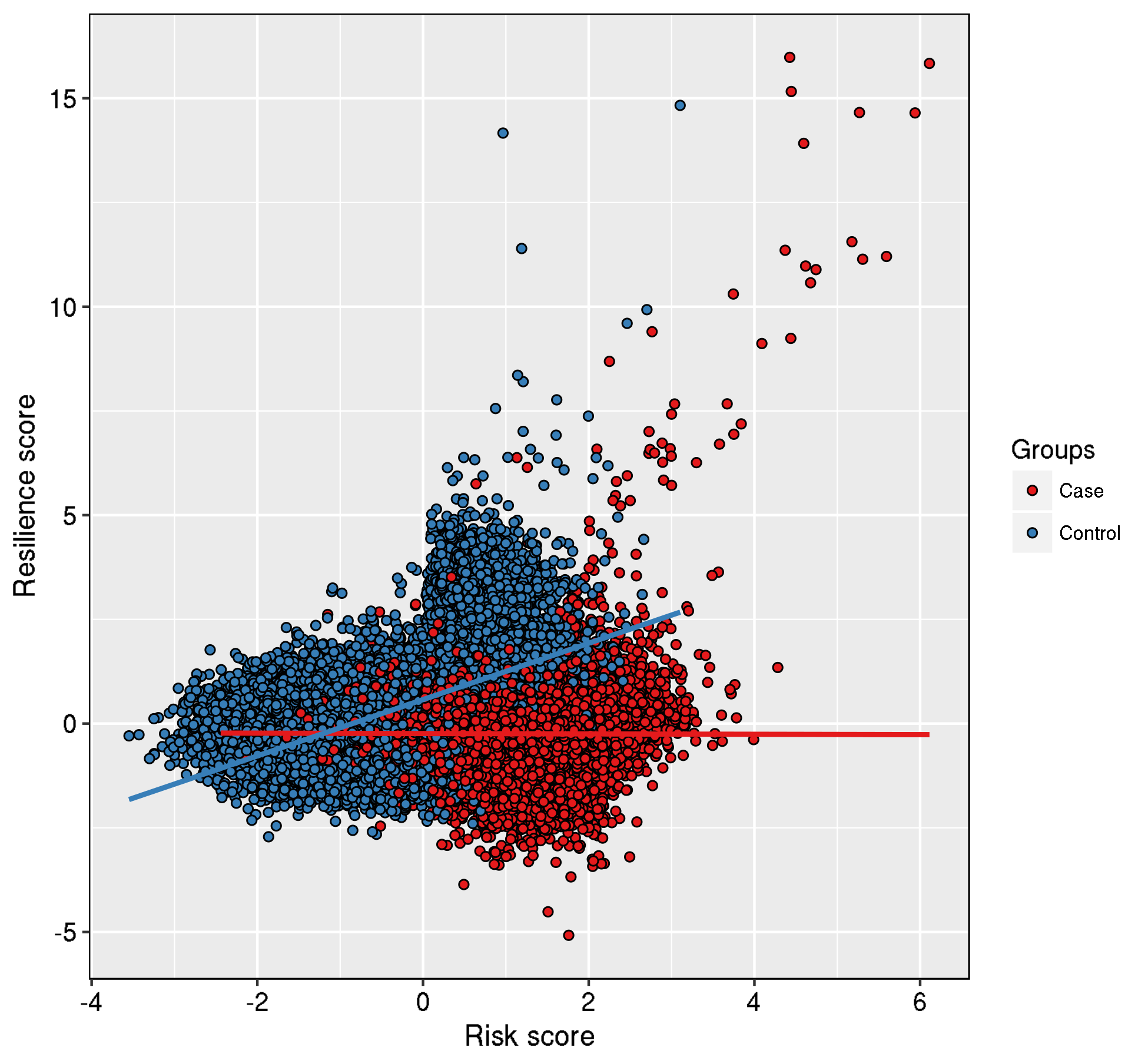


Supplementary Figure 3. Correlation between risk and resilience scores in SZ cases (*n* = 28,869) and controls (*n* = 37,748) in our discovery sample (PGC data set).

Supplementary Table 1**.** Parameters for individual steps of the analysis.

| **Analysis step** | **Parameter** | **Value** | **Justification** |
| --- | --- | --- | --- |
| GWAS sample | Polygenic risk scoring (PRS) | p < 0.05 | Optimal threshold referenced in Ripke et al., (2014) |
|  | PRS threshold for high risk 'resilient' controls | Upper 10% | Arbitrary |
| Reporting GWAS results | MAF filter for SNPs (based on 1000G European panel) | ≥ 5% | Standard cut-off for common variants |
|  | Imputation filter for SNPs | ≥ 0.6 | PGC standard referenced in Ripke et al. (2014) |
| Formulating SNP set for resilience scoring | Imputation filter for SNPs | ≥ 0.9 | PGC standard referenced in Ripke et al. (2014) |
|  | Type of variants removed | Insertions/deletions | PGC standard referenced in Ripke et al. (2014) |
|  | Loci discarded | xMHC region (chr 6:25mb-34mb) | PGC standard referenced in Ripke et al. (2014) |
|  | MAF filter for SNPs | ≥ 5% | Standard cut-off for common variants |
|  | Prune and clump SNPs | --clump-p1 1.0 --clump-p1 1.0 --clump-kb 500 --clump-r2 0.2 | PGC standard referenced in Ripke et al. (2014) |

Supplementary Table 2. The number of resilient controls and high-risk cases identified in each of the 45 cohorts from the Psychiatric Genomics Consortium (PGC).

| **Discovery cohorts** | |  |  |  |  |  |
| --- | --- | --- | --- | --- | --- | --- |
| Study ID | Resilience controls | Total controls | % controls retained | High-risk cases | Total cases | % cases retained |
| aarh | 87 | 871 | 9.99 | 541 | 876 | 61.76 |
| aber | 70 | 697 | 10.04 | 508 | 719 | 70.65 |
| ajsz | 160 | 1,594 | 10.04 | 605 | 894 | 67.67 |
| asrb | 29 | 287 | 10.1 | 292 | 456 | 64.04 |
| boco | 216 | 2,161 | 10 | 1,037 | 1,773 | 58.49 |
| buls | 61 | 608 | 10.03 | 134 | 195 | 68.72 |
| butr | 61 | 613 | 9.95 | 379 | 608 | 62.34 |
| cati | 21 | 203 | 10.34 | 192 | 397 | 48.36 |
| caws | 29 | 284 | 10.21 | 217 | 396 | 54.8 |
| cims | 7 | 65 | 10.77 | 17 | 67 | 25.37 |
| clm2 | 408 | 4,085 | 9.99 | 2,546 | 3,426 | 74.31 |
| clo3 | 198 | 1,975 | 10.03 | 1,765 | 2,105 | 83.85 |
| cou3 | 68 | 678 | 10.03 | 215 | 530 | 40.57 |
| denm | 46 | 456 | 10.09 | 226 | 471 | 47.98 |
| dubl | 84 | 839 | 10.01 | 162 | 264 | 61.36 |
| edin | 29 | 284 | 10.21 | 242 | 367 | 65.94 |
| egcu | 115 | 1,152 | 9.98 | 141 | 234 | 60.26 |
| ersw | 32 | 319 | 10.03 | 132 | 265 | 49.81 |
| fi3m | 93 | 929 | 10.01 | 117 | 186 | 62.9 |
| fii6 | 109 | 1,082 | 10.07 | 240 | 360 | 66.67 |
| gras | 117 | 1,169 | 10.01 | 620 | 1,067 | 58.11 |
| hok2 | 202 | 2,018 | 10.01 | 327 | 476 | 68.7 |
| irwt | 101 | 1,006 | 10.04 | 810 | 1,291 | 62.74 |
| jpn1 | 43 | 427 | 10.07 | 227 | 492 | 46.14 |
| lacw | 25 | 245 | 10.2 | 82 | 157 | 52.23 |
| lemu | 18 | 177 | 10.17 | 94 | 197 | 47.72 |
| lie2 | 27 | 269 | 10.04 | 62 | 133 | 46.62 |
| lie5 | 39 | 389 | 10.03 | 342 | 497 | 68.81 |
| msaf | 14 | 139 | 10.07 | 147 | 325 | 45.23 |
| munc | 32 | 312 | 10.26 | 222 | 421 | 52.73 |
| pewb | 180 | 1,812 | 9.93 | 388 | 574 | 67.6 |
| pews | 24 | 236 | 10.17 | 77 | 150 | 51.33 |
| port | 22 | 215 | 10.23 | 100 | 346 | 28.9 |
| s234 | 228 | 2,274 | 10.03 | 1,387 | 1,980 | 70.05 |
| swe1 | 21 | 210 | 10 | 96 | 215 | 44.65 |
| swe5 | 258 | 2,581 | 10 | 1,286 | 1,764 | 72.9 |
| swe6 | 115 | 1,145 | 10.04 | 585 | 975 | 60 |
| tcr1 | 94 | 938 | 10.02 | 621 | 868 | 71.54 |
| top8 | 41 | 403 | 10.17 | 214 | 377 | 56.76 |
| ucla | 61 | 607 | 10.05 | 501 | 700 | 71.57 |
| uclo | 49 | 485 | 10.1 | 286 | 509 | 56.19 |
| uktr | 4 | 38 | 10.53 | 21 | 42 | 50 |
| umeb | 58 | 577 | 10.05 | 245 | 341 | 71.85 |
| umes | 71 | 704 | 10.09 | 116 | 193 | 60.1 |
| zhh1 | 19 | 190 | 10 | 55 | 190 | 28.95 |
| Total | 3,786 | 37,748 |  | 18,619 | 28,869 |  |

Supplementary Table 3**.** The incidence, absolute risk, and relative risk of SZ calculated after incrementally relaxing the percentile threshold from the 90^th^ percentile down to the 10^th^ percentile. Subjects above the 10^th^ percentile were used as a baseline to calculate SZ risk for subjects in the other percentiles.

| **Percentile threshold** | **Cases (n)** | **Controls (n)** | **Incidence of SZ** | **Absolute increase in risk** | **Relative risk** |
| --- | --- | --- | --- | --- | --- |
| 10 | 26,117 | 33,952 | 0.43 | 0.00 | 1.00 |
| 20 | 26,047 | 29,911 | 0.47 | 0.03 | 1.07 |
| 30 | 25,912 | 26,409 | 0.50 | 0.06 | 1.14 |
| 40 | 25,703 | 22,631 | 0.53 | 0.10 | 1.22 |
| 50 | 25,323 | 18,858 | 0.57 | 0.14 | 1.32 |
| 60 | 24,732 | 15,093 | 0.62 | 0.19 | 1.43 |
| 70 | 23,818 | 11,331 | 0.68 | 0.24 | 1.56 |
| 80 | 22,135 | 7,559 | 0.75 | 0.31 | 1.71 |
| 90 | 18,619 | 3,786 | 0.83 | 0.40 | 1.91 |

Supplementary Table 4. Results from two-sample *t*-tests that compared high-risk “resilient” controls and matched-risk cases from the PGC2-SZ data set on the top 20 principal components for ancestry derived from genome-wide SNP genotypes.

| **Principal component** | **T-statistic** | **P-value** |
| --- | --- | --- |
| C1 | -0.370 | 0.711 |
| **C2** | **-14.347** | **9.50E-46** |
| C3 | -1.132 | 0.258 |
| C4 | 0.414 | 0.679 |
| **C5** | **1.905** | **0.057** |
| **C6** | **-3.884** | **1.04E-04** |
| **C7** | **-1.911** | **0.056** |
| C8 | 0.674 | 0.500 |
| C9 | -0.254 | 0.800 |
| C10 | 0.675 | 0.500 |
| C11 | 0.958 | 0.338 |
| C12 | -0.514 | 0.607 |
| C13 | -1.416 | 0.157 |
| C14 | -0.903 | 0.366 |
| C15 | 1.638 | 0.102 |
| C16 | 0.591 | 0.555 |
| C17 | -0.374 | 0.708 |
| C18 | 1.199 | 0.231 |
| C19 | 0.526 | 0.599 |
| C20 | -0.634 | 0.526 |

Supplementary Table 5. Difference in resilience scores between sub-samples in the discovery and replication sets after principal component ancestry adjustment.

| **Cohorts** | **Subsamples** | **N** | **Beta** | **SE** | ***p*-value** |
| --- | --- | --- | --- | --- | --- |
| Discovery sample | Controls v. cases | 66617 | 0.44 | 0.008 | < 2.2×10^-308^ |
|  | High risk controls v. High risk cases | 22405 | 3.20 | 0.013 | < 2.2×10^-308^ |
|  | High risk controls vs. low risk controls | 37748 | 2.82 | 0.011 | < 2.2×10^-308^ |
| MGS + iPSYCH + deCODE |  |  |  |  |  |
| (combined *via* meta-analysis) | Controls v. cases | 159003 | 0.02 | 0.010 | 0.013 |
|  | High risk controls v. High risk cases | 9556 | 0.08 | 0.025 | 0.002 |
|  | High risk controls vs. low risk controls | 151418 | 0.05 | 0.010 | 8.3×10^-8^ |

**Note**: P-values reported are reported as < 2.2×10^-308^ if the actual value exceeded the smallest positive value that can be represented by the IEEE-754 double-point precision floating point format.

Supplementary Table 6**.** Decile analysis conducted in the full PGC sample comparing the odds of being a case between bottom decile and higher deciles, and odds of being a control per unit increase in resilience score.

|  |  |  | Risk score | | | Resilience score | | |
| --- | --- | --- | --- | --- | --- | --- | --- | --- |
| Risk score decile | *n* cases | *n* controls | OR^⋈^ | SE | *p*-value | OR* | SE | *p*-value |
| 1 | 83 | 6,326 | 1.00 | - | - | 0.85 | 0.17 | 0.39 |
| 2 | 274 | 6,112 | 3.46 | 0.112 | 1.2e-22 | 0.72 | 0.10 | 1.3e-3 |
| 3 | 609 | 5,786 | 8.19 | 0.11 | 3.8e-70 | 0.78 | 0.07 | 2.2e-4 |
| 4 | 1,030 | 5,352 | 15.17 | 0.11 | 9.1e-122 | 0.66 | 0.06 | 9.5e-14 |
| 5 | 1,811 | 45,76 | 32.57 | 0.10 | 1.2e-203 | 0.71 | 0.05 | 2.2e-14 |
| 6 | 2,791 | 3,605 | 67.38 | 0.10 | 9.3e-298 | 1.2 | 0.03 | 1.3e-6 |
| 7 | 3,669 | 2,718 | 122.34 | 0.10 | <1.0e-300 | 3.0 | 0.03 | 6.3e-247 |
| 8 | 4,634 | 1,756 | 253.42 | 0.11 | <1.0e-300 | 5.9 | 0.05 | 2.8e-291 |
| 9 | 5,311 | 1,080 | 479.72 | 0.11 | <1.0e-300 | 73.1 | 0.19 | 3.4e-117 |
| 10 | 5,932 | 437 | 1481.37 | 0.12 | <1.0e-300 | 44.5 | 0.19 | 2.5e-86 |

Note: Ultra-high risk cases (n = 2,850) were excluded from this analysis of the PGC data set. Deciles were constructed within each study of PGC2-SZ collection.

Parameter estimates yielding a significance value of p < 0.05 are bolded. **Abbreviations:** Odds ratio (OR), standard error (SE)

OR^⋈^: Increase in odds of being a schizophrenia case compared to the lowest decile of risk.

OR*: Odds of being a control per unit increase (+1 standard deviation) in resilience score within the risk score decile.

Supplementary Table 7. GWAS meta-analysis summary statistics for 7 index variants associated with resilience at a suggestive level of significance (p < 1e-05), sorted by p-value.

| **CHR** | **BP** | **SNP** | **Protective allele** | **Alternate allele** | **Freq EUR** | **Freq Controls** | **Freq cases** | **Odds Ratio** | **SE** | **P-value** | **# of studies** | **I^2^** | **Max LD with risk SNP (*r*^2^)** |
| --- | --- | --- | --- | --- | --- | --- | --- | --- | --- | --- | --- | --- | --- |
| 19 | 56985951 | rs66718632 | T | G | 0.13 | 0.148 | 0.134 | 1.192 | 0.038 | 3.01E-06 | 44 | 0.00 | 0.0701 |
| 7 | 38541881 | rs1545145 | T | G | 0.06 | 0.062 | 0.051 | 1.314 | 0.059 | 4.41E-06 | 41 | 0.00 | 0.0063 |
| 13 | 53183862 | rs2768186 | T | G | 0.07 | 0.079 | 0.057 | 1.512 | 0.091 | 5.76E-06 | 16 | 0.00 | 0.089 |
| 9 | 93662681 | rs73650288 | A | G | 0.19 | 0.217 | 0.195 | 1.184 | 0.038 | 7.42E-06 | 29 | 0.00 | 0.086 |
| 4 | 17439720 | rs144981949 | G | T | 0.09 | 0.070 | 0.053 | 1.284 | 0.056 | 8.55E-06 | 36 | 0.00 | 0.029 |
| 2 | 8077246 | rs888133 | A | C | 0.05 | 0.053 | 0.042 | 1.319 | 0.062 | 9.31E-06 | 42 | 8.36 | 0.012 |
| 10 | 17420245 | rs11595156 | C | T | 0.06 | 0.057 | 0.044 | 1.330 | 0.064 | 9.55E-06 | 34 | 22.52 | 0.021 |

**Abbreviations**: Chromosome (CHR), base pair (BP), single nucleotide polymorphism (SNP), Allele frequency in subjects of European ancestry in the 1000 Genomes reference panel (Freq EUR), allele frequency in high-risk controls (Freq Controls), allele frequency in high-risk cases (Freq cases), standard error (SE), index of heterogeneity, *i.e*., percent of variation across studies (I^2^).

Supplementary Table 8. Intragenic SNPs in putative resilience genes (with *p* ≤ 1×10^-5^) and top risk SNPs that mapped to the same genes.

| **Top resilience SNPs** | | | | | | | **Top risk SNP** | | | | |  |
| --- | --- | --- | --- | --- | --- | --- | --- | --- | --- | --- | --- | --- |
| **Chromosome** | **Base pair** | **SNP** | **Protective allele** | **Odds ratio** | **P-value** | **Genes** | **SNP** | **Risk allele** | **Odds ratio** | **P-value** | **Genes** | **LD (*R*^2^)** |
| 19 | 56985951 | rs66718632 | T | 1.192 | 3.01E-06 | *ZNF667* | rs1988918 | T | 1.031 | 7.23E-03 | *ZNF667* | 8.94E-02 |
| 7 | 38541881 | rs1545145 | T | 1.314 | 4.41E-06 | *AMPH* | rs10279199 | T | 1.054 | 1.24E-05 | *AMPH* | 1.01E-03 |
| 2 | 8077246 | rs888133 | A | 1.319 | 9.31E-06 | *AC007464.1* | rs193930 | G | 1.035 | 2.61E-03 | *AC007464.1* | 1.90E-03 |
| 10 | 17420245 | rs11595156 | C | 1.330 | 9.55E-06 | *ST8SIA6* | rs45504992 | T | 1.070 | 1.86E-03 | *ST8SIA6* | 1.68E-02 |

SNPs were mapped to genes and RNAs (i.e., protein-coding genes, miRNA, lincRNA, antisense RNA, snoRNA, scRNA, and pseudogenes) according to HG19 coordinates (Gencode GRCh37.p13). **Abbreviations**: single nucleoide polymorphism (SNP), linkage disequilibrium (LD).
